# Asthma diagnosis and treatment – 1004. The utility of mannitol challenge in the assessment of asthma

**DOI:** 10.1186/1939-4551-6-S1-P4

**Published:** 2013-04-23

**Authors:** Dario Antolin-Amerigo, Maria Jose Sanchez-Gonzalez, Mercedes Rodriguez-Rodriguez, Jose Barbarroja-Escudero, Melchor Alvarez-Mon

**Affiliations:** 1 Servicio De Enfermedades Del Sistema Inmune-Alergia, Hospital Universitario Principe De Asturias, Alcala de Henares-Madrid, Spain

## Background

Mannitol challenge is becoming a supplementary and suitable method for the assessment of asthma, commonly used for the diagnosis of Exercise-Induced Asthma, due to its safety and high specificity, demanding less equipment and time requirement. Some studies show a closer relationship between AHR (Airway Hyperresponsiveness) to mannitol and markers of airway inflammation compared with AHR to methacholine in a selected group of asthmatic subjects. We aimed to describe its suitability in a group of patients who were referred to our outpatient clinic with asthma suspicion.

## Methods

Forty-eight patients over 14 years old from our Allergy Department with asthma symptoms (dyspnoea, cough or wheezing) enrolled in the study. Mannitol was inhaled according to manufacturer instructions (Dr. Anderson 9-Step protocol). The study included skin prick testing with a battery of common aeroallergens and respiratory function tests. All patients gave written informed consent.

## Results

Forty-eight patients (27 female; 21 male) with a mean age of 30.61 years old were enrolled in the study. Forty patients were atopic (95.24%), showing at least one positive skin prick test and/or aeroallergen specific IgE. Two patients did not show any positive skin prick test nor aeroallergen-specific IgE. Twenty-two patients (45.83%) had a positive Mannitol Test with a mean PD15 of 155 mg. Mean Fractional exhaled nitric oxide (FeNO) of the former resulted 36.1 ppb. Six patients (27.27%) exhibited a rapid response to Mannitol challenge and were classified as severe AHR. Five patients (22.72%) showed a moderate response to Mannitol (Steps 5-6). Eleven patients (50%) exhibited a mild response to Mannitol (Steps 7-9). All patients who manifested AHR after Mannitol Test presented a rapid recovery (<10 minutes) to baseline FEV1 after Salbutamol inhalation. The mean time required to complete the test was 18 minutes.

## Conclusions

Mannitol might be considered as a convenient method for AHR diagnosis, due to its good safety and time-saving profile, specificity and relatively low equipment requirement.

